# Visual Function and Driving Performance Under Different Lighting Conditions in Older Drivers: Preliminary Results From an Observational Study

**DOI:** 10.2196/58465

**Published:** 2024-06-26

**Authors:** Jingzhen Yang, Enas Alshaikh, Deyue Yu, Thomas Kerwin, Christopher Rundus, Fangda Zhang, Cameron G Wrabel, Landon Perry, Zhong-Lin Lu

**Affiliations:** 1 Center for Injury Research and Policy at the Abigail Wexner Research Institute Nationwide Children’s Hospital Columbus, OH United States; 2 Department of Pediatrics The Ohio State University Columbus, OH United States; 3 College of Optometry, The Ohio State University Columbus, OH United States; 4 Driving Simulation Laboratory, The Ohio State University Columbus, OH United States; 5 Division of Arts and Sciences, NYU Shanghai Shanghai China; 6 Center for Neural Science and Department of Psychology New York University New York, NY United States; 7 NYU-ECNU Institute of Brain and Cognitive Science at NYU Shanghai Shanghai China

**Keywords:** nighttime driving, functional vision, driving simulation, older drivers, visual functions, photopic, mesopic, glare, driving simulator

## Abstract

**Background:**

Age-related vision changes significantly contribute to fatal crashes at night among older drivers. However, the effects of lighting conditions on age-related vision changes and associated driving performance remain unclear.

**Objective:**

This pilot study examined the associations between visual function and driving performance assessed by a high-fidelity driving simulator among drivers 60 and older across 3 lighting conditions: daytime (photopic), nighttime (mesopic), and nighttime with glare.

**Methods:**

Active drivers aged 60 years or older participated in visual function assessments and simulated driving on a high-fidelity driving simulator. Visual acuity (VA), contrast sensitivity function (CSF), and visual field map (VFM) were measured using quantitative VA, quantitative CSF, and quantitative VFM procedures under photopic and mesopic conditions. VA and CSF were also obtained in the presence of glare in the mesopic condition. Two summary metrics, the area under the log CSF (AULCSF) and volume under the surface of VFM (VUSVFM), quantified CSF and VFM. Driving performance measures (average speed, SD of speed [SDspeed], SD of lane position (SDLP), and reaction time) were assessed under daytime, nighttime, and nighttime with glare conditions. Pearson correlations determined the associations between visual function and driving performance across the 3 lighting conditions.

**Results:**

Of the 20 drivers included, the average age was 70.3 years; 55% were male. Poor photopic VA was significantly correlated with greater SDspeed (*r*=0.26; *P*<.001) and greater SDLP (*r*=0.31; *P*<.001). Poor photopic AULCSF was correlated with greater SDLP (*r*=–0.22; *P*=.01). Poor mesopic VUSFVM was significantly correlated with slower average speed (*r*=–0.24; *P*=.007), larger SDspeed (*r*=–0.19; *P*=.04), greater SDLP (*r*=–0.22; *P*=.007), and longer reaction times (*r*=–0.22; *P*=.04) while driving at night. For functional vision in the mesopic condition with glare, poor VA was significantly correlated with longer reaction times (*r*=0.21; *P*=.046) while driving at night with glare; poor AULCSF was significantly correlated with slower speed (*r*=–0.32; *P*<.001), greater SDLP (*r*=–0.26; *P*=.001) and longer reaction times (*r*=–0.2; *P*=.04) while driving at night with glare. No other significant correlations were observed between visual function and driving performance under the same lighting conditions.

**Conclusions:**

Visual functions differentially affect driving performance in different lighting conditions among older drivers, with more substantial impacts on driving during nighttime, especially in glare. Additional research with larger sample sizes is needed to confirm these results.

## Introduction

Drivers aged 65 years and older face a heightened risk of fatal nighttime crashes, surpassed only by drivers aged under 25 years, based on fatal nighttime crashes per distance driven [1-3]. Anatomical and functional changes in vision due to aging contribute significantly to this elevated risk [4-9], resulting in a 2-4 times greater likelihood of fatal nighttime crashes than daytime incidents [2,5]. Concerns among older drivers regarding nighttime driving include heightened glare from oncoming headlights [10,11], slower recovery times after experiencing glare [12], poor recognition of road signs at night [13], and difficulty differentiating contrast in glare conditions [7,14]. Notably, approximately 1 in 3 older drivers restricts or ceases nighttime driving due to these concerns [15,16]. Moreover, various eye diseases prevalent among older adults, such as cataracts, glaucoma, and age-related macular degeneration, further compromise their ability to drive safely at night [17-19].

Despite these challenges, current clinical examinations of vision primarily concentrate on visual acuity (VA) under daytime photopic lighting conditions, disregarding the mesopic (low lighting) and glare conditions encountered during nighttime driving [20,21]. Additionally, the contrast sensitivity function (CSF) [22,23], which quantifies how contrast sensitivity (1/threshold) changes with spatial frequency, is more closely related to performance in daily visual tasks in general [24,25] and driving in particular [14,26-29]. The visual field map (VFM) is another measure that could offer critical insights into functional vision and performance during nighttime driving. The gap in visual assessment poses a significant issue, especially given the substantial increase in the older adult driving population. In 2020, the 48 million licensed drivers aged over 65 years in the United States represented a 68% increase since 2000 [11]. Projections suggest that by 2050, 1 in every 4 licensed drivers will be aged 65 or older [19,30]. Therefore, understanding how age-related vision changes affect driving performance among drivers aged 65 and older is imperative, especially considering the various lighting conditions they encounter [28,31,32]. This knowledge will pave the way for developing and implementing therapeutic interventions and technological assistance to support these drivers in maintaining their driving privileges and ensuring road safety.

To address this research gap, our pilot study examined the associations between visual function and driving performance, assessed by a high-fidelity driving simulator, among drivers 60 and older across 3 lighting conditions: daytime (photopic), nighttime (mesopic), and nighttime with glare. We hypothesized that drivers with worse visual function, measured by VA, CSF, and VFM, would exhibit worse simulated driving performance, including slower average speed, a greater standard deviation of speed (SDSpeed), a greater standard deviation of lane position (SDLP), and longer reaction times to visual stimuli.

## Methods

### Study Design and Participants

We conducted a pilot study with 20 active drivers aged 60 years or older recruited from the Columbus, Ohio, area. All participants held valid driver’s licenses and were active drivers, defined as those who drove at least once per week. We excluded drivers who drove less than once per week, used visual aid devices beyond habitual correction (eg, bioptic lens), or had conditions preventing them from driving, such as cognitive impairment or eye diseases. Non-English speakers and individuals with a history of severe motion sickness were also excluded. The study data were collected between November 2021 and August 2022.

### Study Procedures

We distributed information about our study through channels that had proven successful in our previous research: (1) a website listing and Facebook advertising via StudySearch, a directory managed by the Ohio State University (OSU) Center for Clinical and Translational Science [33]; (2) ResearchMatch, a registry of volunteers managed by the Vanderbilt Institutes for Clinical and Translational Research, spanning a network of 183 institutions [34]; (3) Twitter and Instagram posts facilitated by Nationwide Children’s Hospital; and (4) study flyers distributed at local community centers. Social media postings or email links directed interested individuals to complete an initial eligibility survey on our study website or to meet our study team over the phone for eligibility screening.

Following expressions of interest, our study coordinator contacted the individuals, rescreened them for eligibility, and obtained signed consent. Subsequently, the study coordinator scheduled an in-person appointment for visual function assessments at the OSU College of Optometry’s Laboratory of Vision Enhancement. Additionally, participants underwent a driving performance assessment on a high-fidelity driving simulator at the OSU Driving Simulation Laboratory. These 2 assessments took place within 1 month of each other.

### Visual Function Test

#### Overview

The visual function test was conducted binocularly with participants wearing the habitual corrective lenses they used for daily driving. The test encompassed 3 fundamental visual functions: VA, CSF, and VFM, measured using the quantitative VA, quantitative CSF (qCSF), and quantitative VFM (qVFM) methods, respectively [35-39]. These methods have used an active learning framework to provide efficient assessments while maintaining high precision and accuracy, and they have been validated in approximately 50 peer-reviewed publications, demonstrating high correlations with conventional visual function tests as well as precision and repeatability across the full spectrum of spatial frequencies [37,40-44]. For example, in the quantitative VA task, participants were instructed to identify 3 high-contrast letters in each trial, with the letter size varying between trials. The same applied to the qCSF task, with the exception that the contrast of letters also varied between letters and trials. During the qVFM task, participants were directed to report a light spot that might have briefly presented inside a circular location cue while keeping their gaze fixed in the center of the display for the duration of the test. The target location and luminance were updated in an adaptive manner. The size of the visual field being evaluated was 48°×48°. Two summary metrics, the area under the log contrast sensitivity function (AULCSF) and the volume under the surface of VFM (VUSVFM), scored the CSF and VFM tests. The tests were conducted in 2 or 3 lighting conditions, described in the following sections.

##### Photopic Condition

Background luminance was set at 9 cd/m^2^ or above for all the photopic tests, encompassing VA, CSF, and VFM measurements. A brief practice block was given for each task. A minimum of 5 minutes of dark adaptation was given before starting the first practice.

##### Mesopic Condition

Background luminance was reduced to between 0.1 and 1 cd/m^2^ using a neutral density filter or display control, yielding reliable mesopic measurements. After more than a 5-minute dark adaptation period, participants underwent measurements for mesopic VA, CSF, and VFM using the same methods as photopic assessments.

##### Mesopic With Glare Tests

Mesopic VA and CSF were measured in the presence of glare. An external light source, a Fiilex V70 lamp with an attached dome diffuser, generated glare. The light was placed at the participant’s eye level, with 19 cm in front and 12 cm to the left of the midpoint of the participant’s 2 eyes. The glare source had a color temperature of 3000 K and provided an illumination level of 305 lux, similar to home or office desk lighting (30-1000 lux) [45].

VA was always measured first for each lighting condition, followed by CSF, with VFM as the last measurement taken under photopic and mesopic conditions. Thus, 8 visual function tests were performed, generating VA, AULCSF, and VUSVFM values across 3 lighting conditions. Low VA values and high AULCSF and VUSVFM values indicated better visual function.

### Driving Performance Test

#### Driving Simulator

**Figure 1:**
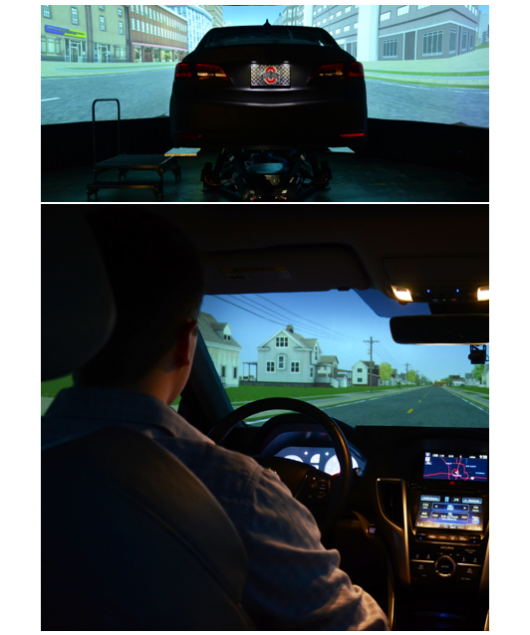
A high-fidelity driving simulator.

The driving performance test on a cutting-edge real-time technology driving simulation platform and lasted approximately 1 hour (Figure 1). This system features a vehicle cab mounted on a 6-degree freedom of motion base, a 260° front-projection cylindrical edge-blended screen, side mirror LCDs, and a rear mirror screen. The custom-developed driving scenarios consisted of driving on a straight, suburban roadway with a posted speed of 72 km/h.

#### Three Experimental Lighting Conditions

We used 3 experimental lighting conditions to provide participants with scenarios and experiences in a safe, controlled environment [27,46,47]. Participants drove with 3 conditions in a random sequence: a *daytime condition* which met the recommended photopic lighting requirements with lighting in the scene coming from headlights from the participant vehicle, a *nighttime condition* which was created by dimming the light in the scene coming from the participant’s vehicle headlights to meet the mesopic light level, and *nighttime with glare condition* which used a point glare source (ie, a lamp fixed to the hood of the car) positioned on the left side of the car simulating headlight glare from oncoming vehicles, allowing the participants to experience the simulated bright headlights shining at the driver from cars in the opposing travel direction. The lamp used was a 650-lumen light-emitting diode bulb positioned approximately 2.1 meters away from the driver’s eye point, 4 degrees to the driver's left.

#### Simulator Procedure

Upon arrival at the OSU Driving Simulation Laboratory, participants completed a 10-minute paper survey about their demographics, motion sickness, history of eye disease, driving habits, and functional impairments before the driving assessment. We advised participants to inform staff if they experienced motion sickness, dizziness, or discomfort while driving on the simulator and to stop the assessment if necessary.

Participants drove a total of 35 minutes in the driving simulator. Following a 5-minute acclimation period, participants completed 3 drives, 1 for each lighting condition, each lasting approximately 10 minutes. The drive sequence was shuffled per participant for counterbalancing. We asked participants to drive as they usually would on a 2-way, undivided roadway with 1 lane in each direction of traffic at a speed limit of 72 km/h. Simulated trees, signs, and buildings were presented on both sides of the road. Participants performed general driving maneuvers, including regulating vehicle speed and direction, lane positioning, and maintaining safe following distance, under light traffic in the opposing lane for all the driving scenarios. During the driving scenarios, participants also encountered half-meter-sized boxes appearing randomly above the roadway in different locations: 1 with a vertical stripe and the other with a horizontal stripe. Participants were instructed to press a button on the steering wheel when they saw a box with only a horizontal stripe.

To regulate the driving speed to around 72 km/h, participants were instructed to stay within 30 meters of a lead car. If they went slower than 64 km/h, the lead car would drive ahead; if they moved faster than 80 km/h, the lead vehicle would not accelerate further. This adaptation helped maintain the participants’ speed between 64 and 80 km/h. During all the driving scenarios, participants also completed a cognitive load task [48], which consisted of conversations between an experimenter and the participant about general interest topics, like travel or food. The experimenter spoke to the participants from a control room through an intercom.

#### Driving Performance Outcome Measures

We measured driving performance using 4 primary variables: average speed (km/h), SDSpeed [49], SDLP [50,51], and reaction time [52]. Reaction time is defined as the time between a target box appearing above the roadway and the participant pressing a button on the steering wheel. Only the reaction time for correct responses (horizontal stripe) was included in the analysis. The driving performance-related variables were measured in multiple periods per drive, surrounding each box appearance, and averaged over the drive. SDSpeed is a measure of the longitudinal control of the vehicle, while SDLP is a measure of the lateral control of the vehicle. SDLP, as a measure of vehicle control in particular, has been used as an indicator of safe driving performance in numerous driving simulation studies [53].

### Other Variables

Gathered demographic information included age, sex, race, marital status, employment, income level, self-assessed overall health, and eye health. Self-reported driving history included average hours driven per day and week and levels of difficulties while driving in various situations in dim light or at night.

### Statistical Analysis

We conducted a descriptive analysis of participants’ demographics and driving variables. We excluded 2 participants from the analyses due to unusable driving data resulting from procedure adjustment or technical difficulties. We used ANOVA, with Bonferroni correction for multiple comparisons, to compare vision scores across 3 lighting conditions. Additionally, we used ANOVA with repeated measures to assess the driving performance variable across 3 lighting conditions. We calculated Pearson correlation coefficients to evaluate the relationships between visual function and driving performance under each lighting condition, and we used the false discovery rate for multiple testing. We conducted all data analyses in SAS and completed the analyses by January 2024, with a significance level of α=.05.

### Ethical Considerations

The study received ethical approval from the Institutional Review Board at Nationwide Children’s Hospital (STUDY00000461). Written informed consent was obtained from all participants. The study was performed in accordance with the ethical standards as laid down in the Declaration of Helsinki and its later amendments or comparable ethical standards and the applicable local regulatory requirements and laws.

## Results

### Characteristics of Participants

**Figure 2:**
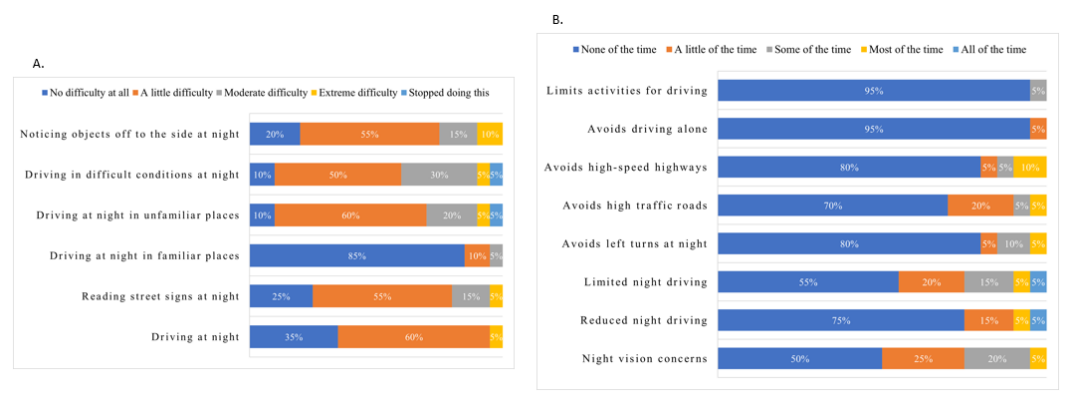
(A) Level of nighttime driving vision difficulties. (B) Frequency of changes in driving habits.

Across all 20 participants, the average age was 70.3 (ranging from 63 to 83), with 45% (n=9) female. A total of 6 participants had had cataract surgery, and 1 participant received a cataract diagnosis but had no surgical intervention. On average, participants had held their driver’s license for about 55 years, and self-reported eyesight averaged 7.6 on a scale of 1-10. In total, 50% (n=10) of the participants reported driving difficulties, ranging from a little difficulty to extreme difficulty, due to concerns about their eyesight in dim light or at night. Additionally, 45% reported driving less in dim light or at night because of their eyesight (Figure 2A). Participants indicated that they avoided driving in dim light or at night due to moderate or extreme difficulties, including driving in difficult conditions (n=8, 40%), driving in unfamiliar places (n=6, 30%), noticing objects off to the side (n=5, 25%), or reading street signs (n=4, 20%) (Figure 2B).

### Visual Functions in 3 Lighting Conditions

The average VA was –0.07 (SD 0.08) in the photopic (daytime) condition, significantly lower (indicating better function) than the average VA in the mesopic (nighttime) condition (mean 0.23, SD 0.11; *P*<.001) and in the mesopic with glare condition (mean 0.44, SD 0.17; *P*<.001) (Table 1). The average AULCSF was 1.51 (SD 0.27) in the photopic condition, significantly greater (indicating better function) than the average AULCSF in the mesopic condition (mean 0.081, SD 0.20; *P*<.001) and in the mesopic with glare condition (mean 0.32, SD 0.21; *P*<.001), which was the worst among the 3 lighting conditions. Finally, the average VUSVFM in the photopic condition was 907.08 (SD 47.05); while lower (indicating worse function) than in the mesopic condition (mean 917.89, SD 78.60; *P*=.60), this difference was statistically insignificant. The lighting conditions accounted for 74%, 83%, and 9% of the variation of VA, AULCSF, and VUSVFM, respectively.

**Table 1:** Visual function scores across 3 different lighting conditions.

Visual function	Overall score (n=60), mean (SD)	Photopic score (n=20), mean (SD)	Mesopic score (n=20), mean (SD)	Glare score (n=20), mean (SD)	*R* ^2^	*P* value^a^
VA^b^	0.2 (0.25)	–0.07 (0.08)	0.23 (0.11)	0.44 (0.17)	0.74	<.001
AULCSF^c^	0.88 (0.54)	1.51 (0.27)	0.81 (0.2)	0.32 (0.21)	0.83	<.001
VUSVFM^d,e^	912.43 (64.16)	907.08 (47.05)	917.89 (78.60)	N/A^f^	0.09	.60

^a^*P* value testing the differences across vision conditions.

^b^VA: visual acuity.

^c^AULCSF: area under the log contrast sensitivity function.

^d^VUSVFM: volume under the surface of the visual field map.

^e^n=40.

^f^N/A: not applicable (VUSVFM was not measured in the presence of glare).

### Driving Performance Under 3 Lighting Conditions

Of the 18 participants included, the average driving speed was 71.55 (SD 8.89) km/h in the daytime condition, significantly slower than that in the nighttime with glare condition (74.60, SD 13.97 km/h) but not significantly different from that in the nighttime without glare condition (73.71, SD 16.33 km/h; *P*=.28) (Table 2). The average SDLP in the nighttime with glare condition was 0.11 (SD 0.07) meters, significantly greater than that in the daytime (mean 0.07, SD 0.04 meters; *P*<.001) and the nighttime without glare condition (mean 0.08, SD 0.04 meters; *P*<.001). Furthermore, the average reaction time was 1.23 (SD 0.29) and 1.21 (SD 0.38) seconds in the nighttime conditions with and without glare, both significantly longer than the average reaction time in the daytime condition (mean 1.07, SD 0.31 seconds; *P*<.001).

**Table 2:** Driving performance scores across 3 different lighting conditions.

Driving performance	Overall score (n=591), mean (SD)	Daytime (n=210), mean (SD)	Nighttime (n=181), mean (SD)	Nighttime with glare (n=200), mean (SD)	*P* value
Average speed (km/h)	73.24 (13.30)	71.55^a^ (8.89)	73.71 (16.33)	74.60 (13.97)	.002
SDSpeed^a^	0.47 (0.61)	0.42 (0.35)	0.51 (0.92)	0.47 (0.47)	.25
SDLP^b^ (meters)	0.08 (0.06)	0.07 (0.04)	0.08 (0.04)	0.11^c,d^ (0.07)	<.001
Reaction time^e^ (seconds)	1.16 (0.34)^f^	1.07^c,g^ (0.31)^h^	1.21 (0.38)^i^	1.23 (0.29)^j^	<.001

^a^SD of speed.

^b^SDLP: SD of lane position.

^c^Significant differences between daytime and nighttime with glare.

^d^Significant differences between nighttime and nighttime with glare.

^e^Time between a target box with a stripe appearing above the roadway and the participant pressing a button on the steering wheel for a correct response (horizontal stripe).

^f^n=448.

^g^Significant differences between daytime and nighttime.

^h^n=182.

^i^n=138.

^j^n=128.

### Correlations Between Visual Function and Driving Performance in 3 Lighting Conditions

**Figure 3:**
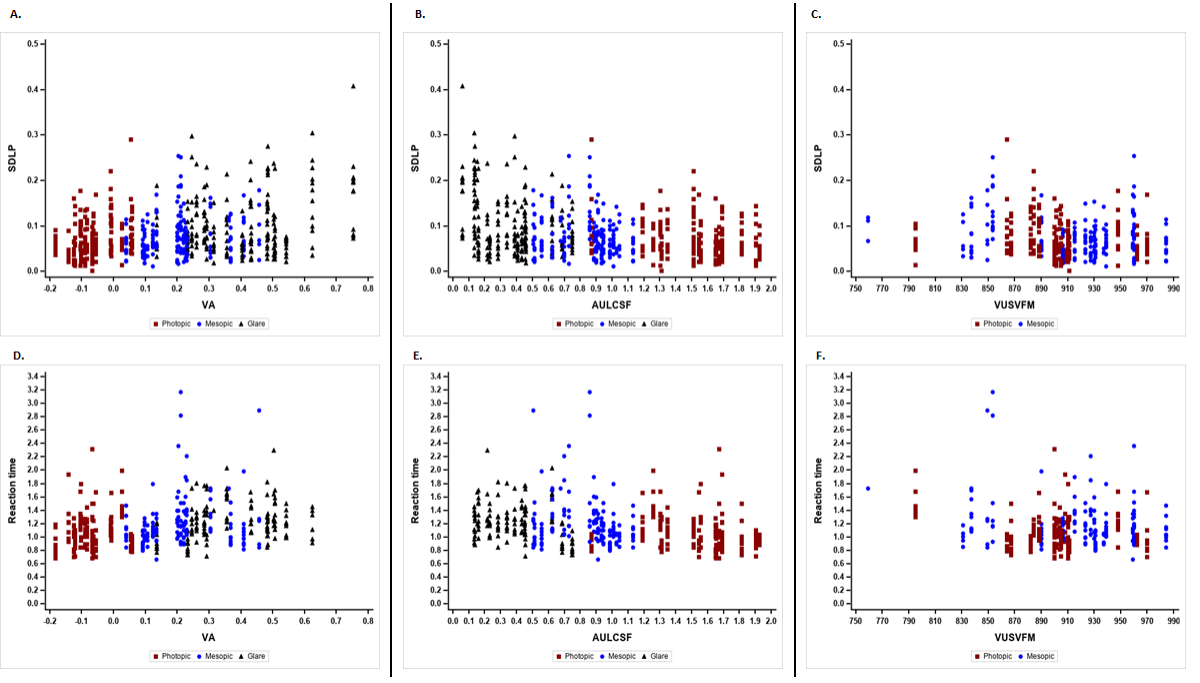
Correlations between visual function and driving performance in 3 lighting conditions. (A) Correlations of VA with SDLP. (B) Correlations of AULCSF with SDLP. (C) Correlations of VUSVFM with SDLP. (D) Correlations of VA with reaction time. (E) Correlations of AULCSF with reaction time. (F) Correlations of VUSVFM with reaction time. SDLP: SD of lane position; VA: visual acuity; AULCSF: area under the log contrast sensitivity function; VUSVFM: volume under the surface of the visual field map.

For functional vision in the photopic condition (Table 3), poor VA was significantly correlated with greater SDspeed (*r*=0.26; *P*<.001) and greater SDLP (*r*=0.31; *P*<.001) (Figure 3A). Poor AULCSF was associated with greater SDLP (*r*=–0.22; *P*=.007) (Figure 3B).

**Table 3:** Pearson correlation coefficients between visual function and driving performance scores across 3 lighting conditions.

Visual function	Daytime (photopic) correlation coefficients					Nighttime (mesopic) correlation coefficients					Nighttime with glare (glare) correlation coefficients			
	Average speed	SDSpeed^a^	SDLP^b^	Reaction time^c^	Average speed		SDSpeed	SDLP	Reaction time^c^	Average Speed		SDSpeed	SDLP	Reaction time^c^
VA^d^	0	0.26^e^	0.31^e^	0.08	–0.03		0.08	0.13	0.17	0.12		–0.04	0.16	0.21^f^
AULCSF^g^	0.08	0.05	–0.22^e^	–0.12	0.09		–0.08	–0.12	–0.17	–0.32^e^		–0.09	–0.26^e^	–0.20^f^
VUSVFM^h^	–0.03	–0.05	–0.13	–0.16	–0.24^d^		–0.19^e^	–0.22^e^	–0.22^e^	N/A^i^		N/A	N/A	N/A

^a^SDSpeed: SD of speed.

^b^SDLP: SD of lane position.

^c^Time between a target box with a stripe appearing above the roadway and the participant pressing a button on the steering wheel for a correct response (horizontal stripe).

^d^VA: visual acuity.

^e^*P* value <.01.

^f^*P* value <.05.

^g^AULCSF: area under the log contrast sensitivity function.

^h^VUSVFM: volume under the surface of the visual field map.

^i^N/A: VUSVFM was not measured in the presence of glare.

For functional vision in the mesopic condition (Table 3), poor VUSFVM was significantly correlated with slower average speed (*r*=–0.24; *P=*.007) and larger SDSpeed (*r*=–0.19; *P*=.04), greater SDLP (*r*=–0.22; *P*=.01) (Figure 3C), and longer reaction times (*r*=–0.22; *P*=.04) (Figure 3F) while driving at night.

For functional vision in the mesopic condition with glare (Table 3); *P*oor VA was significantly correlated with longer reaction times (*r*=0.21; *P*=.046) while driving at night with glare (Figure 3D). Poor AULCSF was significantly correlated with slower speed (*r*=–0.32; *P*<.001), greater SDLP (*r*=–0.26; *P*<.001) (Figure 3B), and longer reaction times (*r*=–0.2; *P*=.04) (Figure 3E) while driving at night with glare. No other significant correlations were observed between visual function and driving performance under the same light conditions.

Furthermore, poor photopic VA was significantly correlated with greater SDLP while driving at night with (*r*=0.25; *P*<.001) and without (*r*=0.28; *P*<.001) glare, respectively [see Table S1 in Multimedia Appendix 1]. Poor photopic AULCSF was significantly correlated with greater SDLP across the 3 lighting conditions, longer reaction times (*r*=–0.24; *P*<.001) while driving at night, and larger SDSpeed (*r*=–0.41; *P*<.001) while driving at night with glare. Poor photopic VUSFVM was significantly correlated with a larger SDLP (*r*=–0.25; *P*<.001) while driving at night with glare. For functional vision in the mesopic condition, poor VA, AULCSF, and VUSFVM were significantly correlated with greater SDLP and/or longer reaction times while driving at night with or without glare. For functional vision in the mesopic condition with glare, poor VA and AULCSF were correlated with longer reaction times in all 3 lighting conditions.

## Discussion

### Key Findings

This pilot study examined the relationships between visual function and driving performance across 3 lighting conditions among drivers aged 60 years and older. The main findings highlight that worse visual function and driving performance were observed in the nighttime condition relative to the daytime condition, particularly with glare. Poor visual functions were largely correlated with poor driving performance. Specifically, poor photopic VA and CSFs were significantly associated with greater SDLP (poor lane keeping ability) while driving in all 3 lighting conditions. Poor mesopic VA, CSFs, and VFM were significantly correlated with greater SDLP and longer reaction times while driving at night with or without glare. Poor VA and CSFs under mesopic conditions with glare were significantly correlated with longer reaction times in all 3 lighting conditions. Our results contribute to the current literature on the relationships between visual functions and nighttime driving and have potential implications for the nighttime driving safety of over 45 million licensed drivers who are aged 65 years and older [11].

Crash statistics indicate that the nighttime driving environment is dangerous for all road users, particularly older drivers [19,54]. Our findings also reveal that participants’ SDLP was significantly greater at night with glare than in the other 2 lighting conditions. Participants also took longer to respond to stimuli (eg, reacting to the boxes appearing above the roadway) during both nighttime conditions compared to the daytime condition. The observed decline in driving performance among older drivers during the nighttime could be attributed to reduced visibility [55] and a combination of age-related vision changes, including reductions in rod sensitivity, slower dark adaptation, decreased VA, and increased sensitivity to glare [5,6,8,9]. All these factors contribute to an increased risk of nighttime crashes [5,53]. Given that access to transportation by car is crucial for the well-being and independence of many older individuals, future studies using comprehensive vision tests are imperative. Such studies can help identify robust and unambiguous indicator(s) of older individuals’ fitness to drive at night, ensuring their driving privileges and road safety [7,19,56,57].

Consistent with previous findings [5-7,28], this study reveals that poor VA and poor CSF were associated with increased SDLP across all 3 lighting conditions—an indicator of poor vehicle control. Moreover, this study found that individuals with poor VA, CSF, and VFM may face greater challenges in maintaining a stable lateral position on the road and take a relatively long time to respond to an event critical to driving safety, particularly while driving at night. These results suggest that compromised visual functions may lead to driving challenges at night, particularly with glare [6]. Therefore, assessing VA alone during daylight, as current standard vision tests do, without considering the effect of nighttime vision or glare, may inaccurately characterize functional vision [6].

Puell et al [14] observed that reduced contrast sensitivity, especially in nighttime conditions with glare, might contribute to challenges during night driving. Wood and Owens [6] found that visibility significantly diminishes during night driving, with contrast sensitivity tests better predicting real-world object recognition performance than VA alone. This study also found that visual function in the mesopic and mesopic with glare conditions was significantly associated with driving performance at night, particularly with glare conditions. Poor vision in low-light conditions is correlated with poor recognition of traffic signs, as low lighting reduces the reading distance of road signs, leaving less time to react. These effects are especially pronounced in older drivers who drive at night [7]. Our results, along with those from other studies [6,7,14], suggest that assessing VA, along with CSF, VFM, and other visual functions, is crucial for characterizing age-related vision changes when evaluating the relationships between vision and driving. These metrics could also serve as targets for interventions to mitigate driving challenges, especially at night, among older adults [27,29].

This pilot study is the first to assess the relationships between visual function and driving performance under different lighting conditions among drivers aged 60 years and older. Understanding the impacts of age-related visual deficits on nighttime driving is critically needed because age-related change in vision is the most significant risk factor for crash-related injuries and deaths at night in older individuals [4-9]. Future research and practical applications could explore developing targeted strategies for addressing specific visual impairments in older drivers in the context of distinct lighting challenges [13,20]. These strategies include considering using polarizing eyeglasses or other glare mitigation techniques [58]. Future studies should also advocate for including novel vision tests that mimic the effects of nighttime conditions and a glare source [5,27,48]. Such assessments may become imperative due to demographic changes (ie, longer life expectancy) compounded by increased dependency on private car journeys among older people [59,60].

### Limitations

This study has several limitations. First, the small sample size and the absence of a control group, along with the lack of data on participants’ other health-related conditions (eg, comorbidities), restricted our ability to conduct more advanced statistical analyses. Adjusting for potential known confounding factors, such as participant age, motor function, and cognitive capacity, was limited. Second, our results may be susceptible to selection bias, possibly reflecting a more active or healthier subset of older drivers. Therefore, caution is needed when generalizing our findings to all drivers aged 60 years and older. Third, the driving measured in this study relied on simulated driving performance in a high-fidelity driving simulator, which may not fully reflect participants’ real-world driving behaviors. Consequently, a cautious interpretation of our results is necessary. Fourth, the participants completed all visual function tests while sitting in a stable chair rather than in a moving vehicle. Thus, the observed relationships between visual function and driving performance in this study may be under- or overestimated. Despite these limitations, our study contributes empirical data on visual function and driving across 3 lighting conditions. We used a set of comprehensive and sensitive visual function measures coupled with simulated driving performance assessed on a high-fidelity driving simulator.

### Conclusions

Drivers aged 60 years and older face a heightened risk of fatal nighttime crashes, primarily attributed to age-related vision changes. This pilot study presents preliminary data indicating that VA, contrast sensitivity, and VFM may distinctly impact driving performance in various lighting conditions among older drivers, particularly at night and in the presence of glare. Further research with a larger and more diverse sample is essential to validating these results. Our results underscore the importance of comprehensive visual function assessments, especially under nighttime and glare conditions, for evaluating age-related vision changes. Additionally, the study findings advocate for developing therapeutic interventions and technological aids to enhance safe nighttime driving among older drivers. As the older adult population is expected to nearly double by 2050, increased research efforts in this domain are warranted to guide clinical practices and policies, ensuring the maintenance of driving privileges and road safety for individuals aged 60 years and older.

## Appendix

Multimedia Appendix 1 Table S1. Pearson correlation coefficients between visual function and driving performance scores across three lighting conditions.

## Abbreviations

AULCSF: area under the log contrast sensitivity function
CSF: contrast sensitivity function
OSU: Ohio State University
SDSpeed: SD of speed
SDLP: SD of lane position
VA: visual acuity
VFM: visual field map
VUSVFM: volume under the surface of visual field map
qCSF: quantitative contrast sensitivity function test
qVFM: quantitative visual field map test

## Data Availability

The data sets generated or analyzed during this study are available from the corresponding author on reasonable request.
