# Supplementary material for: Visual Function and Driving Performance Under Different Lighting Conditions in Older Drivers: Preliminary Results From an Observational Study
Source: JMIR Form Res. 2024 Jun 26;8:e58465. doi: 10.2196/58465 (PMC11237778; doi:10.2196/58465)
Supplement: Multimedia Appendix 1 [file formative_v8i1e58465_app1.docx]

**Appendix 1**

**Table S1. Pearson correlation coefficients between visual function and driving performance scores across three lighting conditions**

|  |  | **Daytime (Photopic)** | | | | **Nighttime (Mesopic)** | | | | **Nighttime with Glare (Glare)** | | | |
| --- | --- | --- | --- | --- | --- | --- | --- | --- | --- | --- | --- | --- | --- |
| **Vision Function** | | **Average Speed** | **SDSpeed** | **SDLP** | **Reaction Time^a^** | **Average Speed** | **SDSpeed** | **SDLP** | **Reaction Time^a^** | **Average Speed** | **SDSpeed** | **SDLP** | **Reaction Time^a^** |
| **VA** | Photopic | 0.00 | 0.26^**^ | 0.31^**^ | 0.08 | 0.20^*^ | 0.22^*^ | 0.28^**^ | 0.21^*^ | 0.12 | 0.10 | 0.25^**^ | 0.07 |
|  | Mesopic | -0.10 | 0.00 | 0.16 | 0.11 | -0.03 | 0.08 | 0.13 | 0.17 | 0.33^**^ | 0.11 | 0.28^**^ | 0.13 |
|  | Glare | -0.02 | -0.04 | -0.04 | 0.26^**^ | 0.12 | 0.04 | 0.05 | 0.19 | 0.12 | -0.04 | 0.16 | 0.21^*^ |
| **AULCSF** | Photopic | 0.08 | 0.05 | ‑0.22^**^ | -0.12 | 0.07 | -0.15 | ‑0.25^**^ | -0.24^*^ | -0.41^**^ | -0.13 | -0.29^**^ | -0.14 |
|  | Mesopic | 0.13 | 0.05 | -0.14 | -0.11 | 0.09 | -0.08 | -0.12 | -0.17 | -0.33^**^ | -0.12 | -0.25^**^ | -0.17 |
|  | Glare | -0.07 | 0.05 | -0.04 | -0.26^**^ | -0.31^**^ | -0.18^*^ | -0.16 | -0.26^*^ | -0.32^**^ | -0.09 | -0.26^**^ | -0.20 |
| **VUSVFM** | Photopic | -0.03 | -0.05 | -0.13 | -0.16 | -0.14 | -0.11 | -0.07 | -0.15 | 0.01 | 0.03 | -0.25^**^ | 0.15 |
|  | Mesopic | -0.08 | 0.01 | ‑0.22^**^ | -0.22^*^ | -0.24^**^ | -0.19^*^ | -0.22^*^ | -0.22^*^ | -0.04 | 0.07 | -0.29^**^ | 0.08 |

VA: visual acuity; AULCSF: area under the log contrast sensitivity function; VUSVFM: volume under the surface of the visual field map; SDSpeed: Standard Deviation of Speed; SDLP: standard deviation of lane position

^a^: Time between a target box with a stripe appearing above the roadway and the participant pressing a button on the steering wheel for a correct response (horizontal stripe).

^**^ P-value <.01

^*^  P-value <.05
